# Another Clincher

**Published:** 1896-07

**Authors:** 


ANOTHER CLINCHER.——Apropos of "that resolution" which gives as a pretext for recognizing homeopathy the fact that the State Constitution says "no preference shall ever be given by law to any school of medicine," and therefore it is necessary to recognize them "in order to get a constitutional law passed," it is recalled that under the present law,—the act of 1879, the only law we have on the subject of regulating the practice, and which law was secured through the efforts of the State Medical Association,—it is stipulated that all diplomas recognized by examining boards under that law must emanate from colleges *recognized by the American Medical Association!* If this is not a "preference," we hardly see how a straight out bill, as advocated by the writer, providing for a board of regular physicians, could be so construed, it being for the purpose of examining physicians only, and not homeopaths (unless they attempt to practice medicine). The present law (of 1879) clearly "gives preference the Americal Medical Association" (composed entirely of what is derisively called allopaths) contrary to the constitution (?); and yet it is constitutional; has been so pronounced by the courts. We quote the language of Judge Fly, Associate Justice of the Court of Appeals, in the case of *Kennedy vs. Schultz.* This case was decided by Judge King, of the Bexar County Court, and an appeal on writs of error taken; one of the errors assigned being that "the articles 3625 to 3638, inclusive, are unconstitutional, and do not impose the same obligations or burdens equally and uniformly upon the same class of citizens, because the same [articles 3625 to 3638 inclusive] are in conflict with that article in the State Constitution which declares that "The legislature may pass laws prescribing the qualifications of practitioners of medicine in this State, and to punish persons for malpractice: but no preference shall ever be given by law to any school of medicine." (See TEXAS MEDICAL JOURNAL, January, 1896, pages 386 and 387.)

Justice Fly said: "There can be no question of the constitutionality of the statute. * * * We can see no conflict between the statute and the constitution. The conflict set up is, that the statute provides that the board of examiners must be graduates of some medical college 'recognized by the American Medical Association,' and that this Association is composed entirely of adherents to the allopathic school of medicine. This court can not possibly know that to be a fact, and can not, of course, go out into the field of speculation in connection with that subject."

Now, what becomes of the pretext that a law would be unconstitutional unless it provided for recognition of homeopaths?

It is all bosh; there is no kind of justification of or excuse for the action of the Texas State Medical Association in suggesting this to the Legislature; suggesting that it is best to have these fellows stuck on to us. Here is proof that the courts would not regard such a law as we want, and should have, as unconstitutional; this case makes the precedent. The bill should be suppressed.

---

NOT BOUND BY ITS ACTION.—The Texas State Medical Association numbers some four hundred—less than five hundred. The number of physicians in the State is estimated at between four thousand and five thousand. There were not exceeding one hundred and fifty members present at the meeting at Fort Worth, and the adoption by them of the draft of the bill recognizing homeopaths can not be taken as an expression of the sentiment of the profession of the State, any more than the declaration of the three tailors of Threadneedle street that "we are the people" can be accepted as the *vox populi*. The State Medical Association is not fairly representative of the profession, in as much as the profession is not fully organized locally, and members do not represent a constituency, and speak for them, as is done in political bodies, where no one but delegates are allowed a voice. On behalf of the great body of the profession, and of himself, the writer repudiates the action of that body.

---

WE WANT TO KKOW.—When a homeopath undertakes to *practice medicine;* when he abandons,—as they nearly all do, in a tight place,—their ridiculous theory and little pills,—why should he not be examined by a *medical* board? What claim has he to be examined on therapeutics and practice by a homeopath?

---

WHY HAVE A HOMEOPATH on an examining board at all? If they practice homeopathy, we have nothing to do with them; homeopathy is no part of medicine, and nobody cares how much they "practice," so long as they stick to their "little truck." "It is harmless," they admit, and "can't hurt anybody": so—why mention them? But, when they attempt to practice *medicine*—that is a gray horse of another color, and they should come up to the standard of requirements for a medical practitioner.

